# The Relationship Between Thymus and Oncogenesis

**DOI:** 10.1038/bjc.1971.79

**Published:** 1971-12

**Authors:** A. E. Papatestas, K. E. Osserman, A. E. Kark

## Abstract

The records of 1243 patients with myasthenia gravis (M.G.) have been reviewed in a retrospective study of the incidence of extrathymic neoplasms. Ninety-four malignant neoplasms were traced.

The onset of the disease (M.G.) coincided with a marked increase in the incidence of extrathymic neoplasms. The observed number of neoplasms in the year of onset of M.G. was three times higher than the expected in a control group. This was in sharp contrast to the lower than expected incidence in the years preceding the onset of M.G.

The incidence remained at higher than the expected levels throughout the course of the disease in patients who did not undergo thymectomy, while in those patients who had thymectomy the incidence decreased to the levels of the general population after the second postoperative year.

These observations suggest an oncogenic thymic influence. The possibility is discussed of the potential oncogenic role of abnormal clones of immunocompetent small lymphocytes of thymic origin.


### THE RELATIONSHIP BETWEEN THYMUS AND ONCOGENESIS

### A STUDY OF THE INCIDENCE OF NON THYMIC MALIGNANCY IN MYASTHENIA GRAVIS

A. E. PAPATESTAS, K. E. OSSERMAN AND A. E. KARK

*From the Department of Surgery, Medicine and the Myasthenia Gravis Clinic and Research Laboratory, The Mount Sinai Hospital, Mount Sinai School of Medicine of the City University of New York, New York, U.S.A.*

Received for publication June 29, 1971

**SUMMARY.—The records of 1243 patients with myasthenia gravis (M.G.) have been reviewed in a retrospective study of the incidence of extrathymic neoplasms. Ninety-four malignant neoplasms were traced.**

**The onset of the disease (M.G.) coincided with a marked increase in the incidence of extrathymic neoplasms. The observed number of neoplasms in the year of onset of M.G. was three times higher than the expected in a control group. This was in sharp contrast to the lower than expected incidence in the years preceding the onset of M.G.**

**The incidence remained at higher than the expected levels throughout the course of the disease in patients who did not undergo thymectomy, while in those patients who had thymectomy the incidence decreased to the levels of the general population after the second postoperative year.**

**These observations suggest an oncogenic thymic influence. The possibility is discussed of the potential oncogenic role of abnormal clones of immunocompetent small lymphocytes of thymic origin.**

THE involvement of the thymus in the pathogenesis of malignant disease has been suggested experimentally by alterations (increase or decrease) in the incidence of tumor formation following thymectomy (Miller, 1961), and by the clinical observations that thymic pathology is often encountered in immunological disorders commonly associated with extrathymic neoplasms: primary immunological deficiency syndromes (Alexander and Good, 1970), autoimmune diseases (Blumenthal and Berns, 1966; Pirofsky, 1968). These observations have led to the suggestion that persistent thymic abnormalities might be associated with diminished resistance to some types of neoplasia (Miller, 1967).

Thymic pathology is frequently encountered in M.G. (Alpert *et al.*, 1971), a disease considered to be autoimmune in nature. To determine the role of the thymus gland and the effects of thymectomy on human oncogenesis, comparison has been made between the incidence of extrathymic neoplasms in non-thymectomy and in a thymectomy group of myasthenic patients.

Note: Requests for reprints should be addressed to: Dr. A. E. Kark, Department of Surgery, The Mount Sinai Hospital, Fifth Avenue and 100th Street, New York, N.Y. 10029.

### CLINICAL MATERIAL AND METHODS

The records of 1243 patients with myasthenia gravis who have been registered at the Myasthenia Gravis Clinic of the Mount Sinai Hospital (M.S.H.) of New York City, New York, between the years 1951–71, have been reviewed.

The patients were separated into four categories according to the presence or absence of thymomas, and whether or not they had undergone thymectomy (Table I). The following factors pertaining to the occurrence of neoplasia were studied: the crude incidence of malignant neoplasms in each of the four categories, the primary sites, the interval between the diagnosis and the onset of symptoms

TABLE I.—1243 *Patients with Myasthenia Gravis Followed at the Mount Sinai Hospital*

| | No thymectomy | | | Thymectomy | | | All | | |
|---|---|---|---|---|---|---|---|---|---|
| | Men | Women | Total | Men | Women | Total | Men | Women | Total |
| Myasthenia gravis without thymoma | 430 | 532 | 962 | 36 | 111 | 147 | 466 | 643 | 1109 |
| Thymomatous myasthenia gravis | 17* | 38* | 55* | 32 | 47 | 79 | 49 | 85 | 134 |
| | 447 | 570 | 1017 | 68 | 158 | 226 | 515 | 728 | 1243 |

* Includes inoperable thymomas, those treated with irradiation and those diagnosed at autopsy.

TABLE II.—*Person–Years of Observation for the Three Periods at Risk*

| | Period before onset of M.G. | | Period after onset of M.G. (non-thymectomy) | | Post-thymectomy period | |
|---|---|---|---|---|---|---|
| Age groups | Men | Women | Men | Women | Men | Women |
| 0–9 | 4814 | 6988 | 195 | 193 | 3 | 14 |
| 10–19 | 4620 | 6242 | 135 | 491 | 17 | 112 |
| 20–29 | 4202 | 4636 | 198 | 1029 | 78 | 267 |
| 30–39 | 3564 | 3008 | 380 | 1236 | 40 | 243 |
| 40–49 | 2788 | 1806 | 470 | 1049 | 49 | 146 |
| 50–59 | 1950 | 886 | 577 | 660 | 22 | 37 |
| 60–69 | 806 | 392 | 470 | 287 | 21 | 22 |
| 70–79 | 130 | 148 | 211 | 114 | 8 | 4 |
| 80 over | 1 | 10 | 8 | 28 | — | — |
| Total | 22873 | 24116 | 2644 | 5085 | 238 | 845 |

of M.G. and the mortality due to these neoplasms. Adjustments for secular time were not made since over 90% of the neoplasms occurred between 1951–70.

The risk of cancer development (extrathymic neoplasms) in these patients, based on person–years of observation, was assessed in the following three periods:

A. Before the onset of M.G., *i.e.* from birth until the onset of the disease.
B. After the onset of the M.G. (M.G. non-thymectomy period), *i.e.* from the time of onset of myasthenic symptoms until the time of thymectomy, death, loss to, or end of follow-up.
C. Post-thymectomy, *i.e.* from the time of operation until death, loss to, or end of follow-up.

The person–years of observation in each category and for each period were separated into 10 year age groups. The sex and age distribution of the person–years of observation for the three periods are shown in Table II.

The observed numbers of extrathymic neoplasms in the M.G. patients were compared with the expected numbers by applying the general population rates (Cancer registry of Connecticut 1963) specific for sex and age, to the corresponding sex and age groups of M.G. patients, and summing up the expected cases in each category and for each period.

Two additional groups of Mount Sinai Hospital patients were used as control populations: 760 patients admitted for herniorrhaphy (inguinal-femoral-umbilical) in the period 1963–70 and 380 patients admitted for regional enteritis or granulomatous enterocolitis in the period 1955–70.

The risk of cancer development in these groups, based on person–years of observation was assessed separately for the period preceding and following the year of the hospital admission for hernia repair, or the year of admission in which the diagnosis of regional enteritis was first made. A separate assessment was made for that year in both groups. Patients admitted for herniorrhaphy, in whom

TABLE III.—*Primary Site of 94 Extrathymic Neoplasms in Patients with Myasthenia Gravis*

| | |
|---|---|
| Breast | 25 |
| Genital organs | 14 |
| Prostate (5), ovary (3), uterus (4), other (2) | |
| Digestive organs | 12 |
| Colon-rectum (6), pancreas (2), liver (1) stomach and small intestine (2), other (1) | |
| Respiratory system | 9 |
| Lung (8), larynx (1) | |
| Skin | 7 |
| Leukemia | 6 |
| Brain and CNS | 5 |
| Lymphomas | 5 |
| Lymphoma-lymphosarcoma 4 | |
| Multiple myeloma 1 | |
| All other and unspecified | 11 |
| All extrathymic sites | 94 |

hernia repair was postponed because hospital workup revealed underlying neoplasia, were not excluded from the study. The sex and age distribution of the person–years of observation were analyzed in a manner identical to that applied to the M.G. population. The observed annual incidence of neoplasms for each period at risk in the two groups was compared with that expected in a sex and age matched sample of the general population applying the same rates as in M.G. patients.

*Incidence of malignant neoplasms in M.G. patients*

The total number of patients with associated malignancies, thymic or extrathymic was 126, of whom thirteen (10·5%) had multiple malignant neoplasms. The total number of thymic and extrathymic malignant neoplasms was 140 (11% of all patients); forty-six were malignant thymomas and 94 extrathymic tumors. (Eighty-eight additional thymomas classified clinically and pathologically as benign were specifically not included in this study.) Histologic confirmation of malignancy was available in 85 (90%) of the extrathymic neoplasms: in the remainder the histology of the neoplasms was indeterminate.

*Extrathymic malignancies*

An overall incidence of 7·5% of extrathymic malignancies was noted; the incidence was 7·4% for the non-thymomatous and 8·9% for the thymomatous group.

The primary sites are shown in Table III; 31 of these occurred in men and 63 in women. Breast was the most common primary site (40% of all neoplasms in myasthenic women). Frequent occurrence of multiple extrathymic neoplasms was noted in myasthenic women who had not undergone thymectomy (8 patients).

The age of diagnosis of the extrathymic neoplasms is shown in Fig. 1 and compared with the age of onset of M.G. in the 1243 patients. Fig. 2 shows the interval between the diagnosis of the neoplasms and the onset of M.G.

FIG. 1.—Age distribution of onset of M.G. and of extrathymic neoplasms in 1243 patients.

Based on the age and sex distribution of the 46, 989 person–years of observation for the period preceding the onset of M.G. (Table II), the expected number of extrathymic neoplasms is 56; in fact 25 were observed. By contrast, the observed number of neoplasms for M.G. non-thymectomy period (64) was considerably higher than the expected (28). Fig. 3 shows that the annual incidence of neoplasms increased sharply with the onset of M.G. and remained high throughout the duration of the disease in patients who did not undergo thymectomy. Of the 64 neoplasms that occurred in the M.G. non-thymectomy period 39 were in women and 25 in men; the expected figures are 14 and 14 respectively.

Following thymectomy, however, the annual incidence decreased to expected levels (Fig. 4). Four of the five neoplasms that occurred in that period were

INTERVAL BETWEEN DIAGNOSIS OF EXTRATHYMIC MALIGNANT NEOPLASMS AND ONSET OF M.G.

NUMBER OF EXTRATHYMIC NEOPLASMS

Period Prior to the Onset of M.G.

Period of Follow Up After the Onset of M.G.

15 14 13 12 11 10 9 8 7 6 5 4 3 2 1 0 1 2 3 4 5 6 7 8 9 10 11 12 13 14 15 16 17 18 19 20 >20

YEARS

FIG. 2.

ANNUAL INCIDENCE PER 1000 OF EXTRATHYMIC MALIGNANT NEOPLASMS PRIOR TO AND AFTER THE ONSET OF M.G. (NON-THYMECTOMY)
Actual incidence
Expected incidence in age and sex matched group of general population *
ANNUAL INCIDENCE PER 1000
Period Prior to the Onset of M.G.
Period After Onset of M.G.
15 11 10 6 5 1 0 4 5 9 10 14 15 19 YEARS
Onset M.G.
* Computed from the 1963 figures of the Connecticut Cancer Registry

FIG. 3

diagnosed in the first two postoperative years. In the second five-year period after thymectomy, the incidence of neoplasms was not increased.

In the control group of hernia patients, the highest annual incidence of cancer occurred in the year following admission for hernia repair. However, even in that year the actual incidence was only slightly higher than the expected: (actual 5·26‰, expected 5·03‰) in contrast to the three-fold increase that occurred in the M.G. patients (Fig. 3). Furthermore, there was no sharp difference in the actual incidence between the period before, and that following the year of herniorrhaphy. By contrast, in the second control group the incidence of neoplasms before the onset of the regional enteritis and/or granulomatous enterocolitis was below the

FIG. 4.

expected levels, while following the onset of the disease an increase to levels 50% higher than the expected occurred. Comparison of the actual and expected annual incidence of neoplasms for the year following the admission at which the diagnosis of regional enteritis was made, did not reveal a sharp increase as in the M.G. patients.

*Mortality*

Two hundred and seventy-five of the 1243 patients have died (22%). One hundred and seventy-eight deaths (64%) were directly due to M.G. (of which 101 occurred within 3 years of the onset of M.G.) and 10 of these fatalities had associated extrathymic neoplasms. Twenty deaths were of undetermined cause and two fatalities in this group had associated extrathymic neoplasms.

Of the remaining 77 non-myasthenic deaths of known cause (28% of all deaths)

28 were directly attributed to extrathymic neoplasms; all the latter occurred in patients who had not undergone thymectomy.

### DISCUSSION

The effects of thymectomy in human oncogenesis have not been studied, primarily because the operation, except for thymic tumours, has been essentially limited to the treatment of M.G., a rare disease in the general population (incidence: 1 per 18,000) (Keynes, 1969).

The reported incidence of extrathymic neoplasms in patients with M.G. varies between 1·4% and 2·5% and the association has until now been considered to be fortuitous (Lambert and Rooke, 1965). Osserman (1958) reported eight malignancies among 325 patients with myasthenia gravis (2·5%). Lambert and Rooke (1965) reported 22 carcinomas in 857 patients with M.G. seen at the Mayo Clinic (2·5%), while Wolf *et al.* (1966) found six neoplasms in 399 myasthenic patients (1·5%). Other scattered reports of M.G. associated with neoplasms have appeared since 1953 (Anderson *et al.*, 1953; Cohen and Waxman, 1967).

Increased incidence of extrathymic cancer in patients with thymoma regardless of the presence of M.G. has been reported by Souadjian *et al.* (1968). Of interest is Ferguson's (1962) detailed follow-up study over 28 years of 145 cases of M.G. A high mortality from extrathymic neoplasms is evident: 30 deaths occurred in patients who had not undergone thymectomy, 15 due to M.G. and 15 to non-myasthenic causes; of the latter more than half (8) were due to malignant neoplasms (5 breast, 1 bronchus, 1 bladder, 1 gastrointestinal tract). No death was attributed to neoplasia in the thymectomy group.

The frequency of breast cancer is noteworthy. In our series a high incidence was observed in all three risk periods. A separate study on the relationship of breast cancer and thymic abnormalities is to be reported.

The overall incidence of multiple neoplasms in M.G. patients (10·5%) was also higher than the 5·1% reported incidence for multiple primaries (Moertel, 1966), and the ratio of deaths from extrathymic neoplasms to all non-myasthenic deaths was also elevated. All multiple neoplasms and all neoplastic deaths occurred in non-thymectomy patients.

The 7·5% incidence of extrathymic neoplasms in the present series is considerably higher than that previously reported in M.G. patients. This increase has been present in both the thymomatous (8·9%) and the non-thymomatous (7·3%) M.G. patients. The period of highest risk was the one after the onset of M.G. and before thymectomy (observed neoplasms 64, expected 28).

In the period preceding the onset of M.G. the low incidence of neoplasms might be partly attributable to the omission of persons who developed cancer and died, who, had they lived, might have developed M.G. This weakness, however, also exists in any age specific study of the incidence of cancer, in that patients who die from any cause before reaching the age group under study are excluded. In the M.G. non-thymectomy period a similar problem exists: (a) since M.G. has a high mortality, particular in the first three years of the disease, (Papatestas *et al.*, 1971; Simpson, 1958) the non-survivors who might have developed cancer are excluded from the study; (b) more than one-half of the 1243 patients were first seen at Mount Sinai Hospital after the fifth year from the onset of the disease, therefore patients who had cancer and died in the first five years of the disease are also excluded from this sample. For these reasons underestimation of the cancer incidence in the M.G. non-thymectomy period is as probable as in the period before the onset of M.G.

Because of these limitations and for the reasons described below, we have studied the incidence of neoplasms in two control groups. The herniorrhaphy group was included to evaluate whether the annual incidence of cancer in our hospital population undergoing a complete workup on admission and under close observation for a year in follow-up clinic, is significantly higher than that expected in a sex and age matched group of the general population. The differences and the peculiarities in cancer incidence of a hospital population compared with the general population are well known (Lilienfeld *et al.*, 1967). The incidence of cancer in the herniorrhaphy group in the year of admission was only slightly higher than that expected, while a three-fold increase in cancer incidence in the M.G. population for the year of the onset of the disease was observed. Furthermore, in the hernia group there was no difference in the incidence of cancer in the pre- and post-herniorrhaphy periods.

The M.G. and the hernia patients are not strictly comparable populations, some of the former being referred to the hospital from out of state or overseas, while the latter represent a cross section of the population of New York City. Yet the comparison shows that the sharp increase of cancer coinciding with the onset of M.G. probably cannot be attributed only to the close follow-up of a hospital population.

The second control group was chosen to evaluate whether the M.G. pattern of cancer incidence (Fig. 3) is present in other chronic diseases in which autoimmune mechanisms are implicated. A pattern similar to that observed in myasthenic patients was found in that group, *i.e.* increase in cancer incidence coinciding with the onset of the disease. However, the increase over the expected incidence for the period following the diagnosis of regional enteritis was only one-half the observed increase in the M.G. non-thymectomy period. This study is still in progress and will be reported separately in detail.

It would seem, therefore, that the increase of cancer incidence in the M.G. non-thymectomy period (Fig. 3) is not attributable to the bias of a retrospective study, nor to the variations and peculiarities inherent in a study of a hospital population, but is rather a characteristic of the disease itself.

The cancer incidence in the post-thymectomy period showed a marked decline after the second post-operative year. Because of the four neoplasms that occurred during the first two post-operative years and the small number of person–years of observation, the risk in the first five post-operative years remained elevated, while the annual incidence of neoplasms in the second five-years post-thymectomy period fell to the expected levels of the general population.

In a thymectomy series with a longer follow-up (average 12 years) reported by Doll and Kinlen (1970) the cancer incidence was within the expected general population levels.

The findings of an increased incidence during the M.G. non-thymectomy period, together with the observed decrease following thymectomy (Fig. 4), suggests that the abnormal thymus plays an oncogenic role. Other evidence of thymic involvement in human oncogenesis has been provided by (1) the increased incidence of reticuloendothelial malignancies in patients with poorly developed or involuted thymus glands (primary immunological deficiency syndrome: Ataxia Telangiectasia of Louis-Bar and the non-sex linked immunoglobulin deficiencies) (Alexander and Good, 1970); (2) the frequent occurrence of extrathymic neoplasms, particularly of the reticuloendothelial system in patients with autoimmune diseases: rheumatoid arthritis (Blumenthal and Berns, 1966; Goldenberg *et al.*, 1969), Sjorgen's syndrome (Talal and Bunin, 1966), autoimmune anemias (Pirofsky, 1968) and lupus erythematosus (Miller, 1967). Thymic pathology is a common finding in these diseases (Miller, 1965) either in the form of thymic hyperplasia with germinal center formation and/or epithelial, lymphoepithelial or spindle cell thymomas.

The coexistence of immunological deficiencies, autoimmune diseases and neoplasia in patients with thymic pathology has led to the suggestion by Miller (1967) that persistent thymic abnormalities might be associated with diminished resistance to some types of neoplasia. Thomas (1959) postulated that the function of cellular immunity is the recognition and rejection of mutant cells as foreign, and the thymic control mechanism against neoplasia was termed immunosurveillance by Burnet (1967). In Good's view the surveillance system permits cells which are prone to develop malignancy to function to the advantage rather than disadvantage of the host (Good and Finstad, 1968). The suggestion has been made that this immunosurveillant mechanism is mediated by the small lymphocytes of thymic origin which are capable of expressing immune responses (Burnet, 1967). The increased frequency of tumors is a recognized hazard of impaired immunosurveillance (Lawrence, 1970), and the increase of cancer in elderly patients may be attributed to a decreased activity of the thymic immunosurveillance (Walford, 1969).

The clonal theories of autoimmunity (Burnet, 1959, 1965; Burch and Burwell, 1965) have attributed autoimmune manifestations to the proliferation of mutant self-reacting clones of immunocompetent lymphocytes that escape the recognition mechanism of thymic immunosurveillance, thus proliferating and reacting against target organs of the host.

Clinical evidence supporting these theories has been provided by the observations that thymectomy in myasthenia gravis results in stable and permanent remissions, and that the delay in the onset of these remissions is directly related to the activity of the thymic gernimal centers (Papatestas *et al.*, 1971). This observation, originally made on 64 M.G. patients, has been confirmed in a larger series (Perlo *et al.*, in press). Since thymic germinal centers are viewed as possible centers of proliferation of " forbidden " clones (Burnet and Holmes, 1966), the effects of thymectomy in M.G. could be attributed to the elimination of the sites of proliferation of these clones (Papatestas *et al.*, 1971).

Thymectomy in experimental animals is not always followed by an increased incidence of neoplasia; the reduced occurrence of neoplasms such as mammary tumors (Allison and Taylor, 1967; Yunis *et al.*, 1969), lymphomas and lymphoid leukemia (Miller, 1961) following experimental thymectomy suggest that the thymus plays an oncogenic role, in addition to its immunosurveillant one.

It is possible that both these roles are mediated through the small immunocompetent lymphocytes of thymic origin; the allogeneic behaviour of the abnormal self-reacting clones of thymic lymphocytes might well have an oncogenic potential since allogeneic lymphocytes have been shown to promote chromosomal aberrations (Fialkow, 1967) and to induce neoplasia in experimental animals (Schwartz and Beldotti, 1965; Lancet, 1969). Therefore, the increased incidence of neoplasia in autoimmune disease could be linked to the allogeneic behaviour of the self-reacting clones.

The reported frequent occurrence of neoplasms in target organs of autoimmune disorders—thymomas in myasthenia gravis, colonic carcinomas in ulcerative colitis and thyroid carcinomas in thyroiditis (Blumenthal and Berns, 1966)—is consistent with an oncogenic role for the abnormal clones.

In the light of these observations further study of the thymic role in autoimmunity and human oncogenesis is indicated.

The authors are indebted to R. S. Osserman, B.S., for her invaluable help and enthusiastic cooperation in reviewing the patients' records. In particular, our thanks are due to Professor Kurt W. Deuschle, Chairman of the Department of Community Medicine of the Mount Sinai School of Medicine, for advice on the Epidemiological studies.

This work was supported in part by the Rousso and Fins Foundations.

### REFERENCES

Alexander, J. W. and Good, R. A.—(1970) 'Immunobiology for Surgeons'. Philadelphia (Saunders).

Allison, A. C. and Taylor, R. A.—(1967) *Cancer Res.*, **27**, 703.

Alpert, L. I., Papatestas, A. E., Kark, A. E., Osserman, R. S. and Osserman, K. E.—(1971) *Archs Path.*, **91**, 55.

Anderson, R. J., Churchill-Davidson, H. C. and Richardson, A. T.—(1953) *Lancet*, ii, 1291.

Blumenthal, H. T. and Berns, A. W.—(1966) *Gerontological Res.*, **1**, 289.

Burnet, F. M.—(1965) *Br. med. J.*, i, 338.—(1959) 'The Clonal Selection Theory of Acquired Immunity'. London (Cambridge University Press).—(1967) *Lancet*, i, 1171.

Burnet, F. M. and Holmes, M. C.—(1966) *J. Path. Bact.*, **88**, 229.

Burch, P. R. J. and Burwell, R. G.—(1965) *Q. Rev. Biol.*, **40**, 252.

Cancer Registry of Connecticut—(1963) Connecticut State Department of Health, Hartford, Connecticut.

Cohen, S. M. and Waxman, S.—(1967) *Archs intern. Med.*, **120**, 717.

Doll, R. and Kinlen, L.—(1970) *Br. med. J.*, iv, 420.

Ferguson, F. R.—(1962) *Proc. R. Soc. Med.*, **55**, 49.

Fialkow, P. J.—(1967) *Science, N.Y.*, **155**, 1676.

Goldenberg, G. J., Paraskevas, F. and Israels, L. G.—(1969) *Arthritis Rheum.*, **12**, 569.

Good, R. A. and Finstad, J.—(1968) *Trans. Am. clin. clim. Ass.*, **79**, 60.

Keynes, G.—(1969) 'The History of Myasthenia Gravis'. In 'Myasthenia Gravis', edited by R. Greene. Philadelphia (Lippincott).

Lambert, E. H. and Rooke, E. D.—(1965) 'Myasthenic State and Lung Cancer'. In 'The Remote Effects of Cancer on the Nervous Systems Contemporary Neurology Symposia', edited by Lord Brain and F. H. Norris. New York (Grune and Stratton), Vol. 1, p. 67.

*Lancet*—(1969) i, 194.

Lawrence, H. S.—(1970) *New Engl. J. Med.*, **283**, 411.

Lilienfeld, A. M., Pedersen, E. and Doed, J. E.—(1967) 'Cancer Epidemiology: Methods of Study'. Baltimore (Johns Hopkins Press).

Miller, D. G.—(1967) *Ann. intern. Med.*, **66**, 507.

MILLER, J. F. A. P.—(1961) *Adv. Cancer Res.*, **6**, 291.—(1965) ' The Function of the Thymus in Immunity the Scientific Basis of Surgery ', edited by W. T. Irving. London (Churchill), p. 468.—(1967) ' The Thymus in Relation of Neoplasia. Modern Trends in Pathology ', edited by T. Crawford. New York (Appleton Century Crafts), Vol. 2, 140.

MOERTEL, G. G.—(1966) ' Multiple Primary Malignant Neoplasms. Their Incidence and Significance '. New York (Springer-Verlag Inc.).

OSSERMAN, K. E.—(1958) ' Myasthenia Gravis '. New York (Grune and Straton).

PAPATESTAS, A. E., ALPERT, L. I., OSSERMAN, K. E., OSSERMAN, R. S. AND KARK, A. E. —(1971) *Am. J. med.*, **50**, 465.

PERLO, V., ARNASON, B., POSKANZER, D. M., CASTLEMAN, D., SCHWAB, R. S., OSSERMAN, K. E., PAPATESTAS, A. E., ALPERT, L. I. AND KARK, A. E.—(1971) *Ann. N.Y. Acad. Sci.*, **183**, 308.

PIROFSKY, B.—(1968) *Ann. intern. Med.*, **68**, 109.

SCHWARTZ, R. S. AND BELDOTTI, L.—(1965) *Science, N.Y.*, **149**, 1511.

SIMPSON, J. A.—(1958) *Brain*, **81**, 112.

SOUDJIAN, J. B., SILVERSTEIN, M. N. AND TITUS, J. L.—(1968) *Cancer, N.Y.*, **22**, 1221.

TALAL, N. AND BUNIN, J. J.—(1966) *Am. J. Med.*, **36**, 529.

THOMAS, L.—(1959) Discussion of Medawar P.B.: 'Reactions to homologous tissue antigens in relation to hypersensitivity, cellular and humoral aspects of the hypersensitive states', edited by H. S. Lawrence. New York (Harper Medical Division, Harper and Row) p. 529.

WALFORD, R. L.—(1969) ' Etiology and Pathogenesis of Aging, Consideration from an Immune Standpoint, the Immunologic Theory of Aging '. Baltimore (Williams and Wilkins), p. 137.

WOLF, S. M., ROWLAND, L. P. AND SCHOTLAND, D. L.—(1966) *Ann. N.Y. Acad. Sci.*, **135**, 517.

YUNIS, E. J., MARTINEZ, C., SMITH, J., STUTMAN, O. AND GOOD, R. A.—(1969) *Cancer Res.*, **29**, 174.


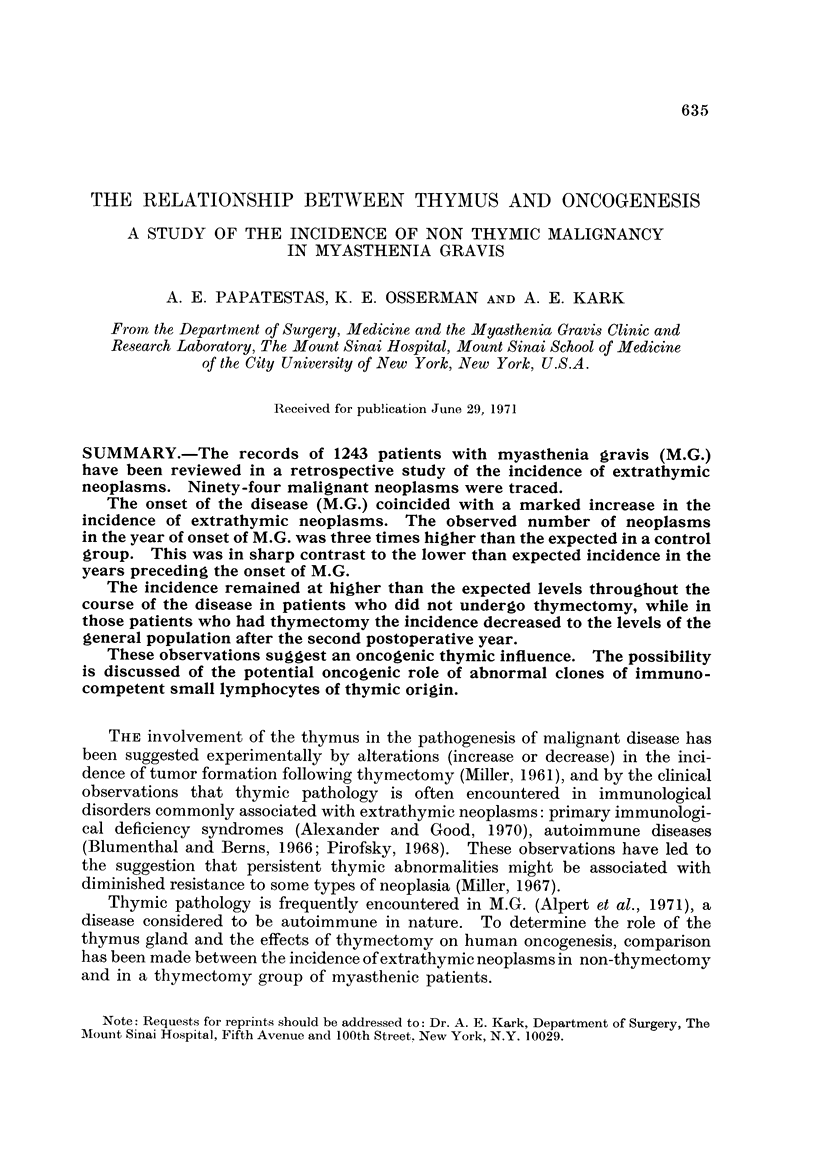

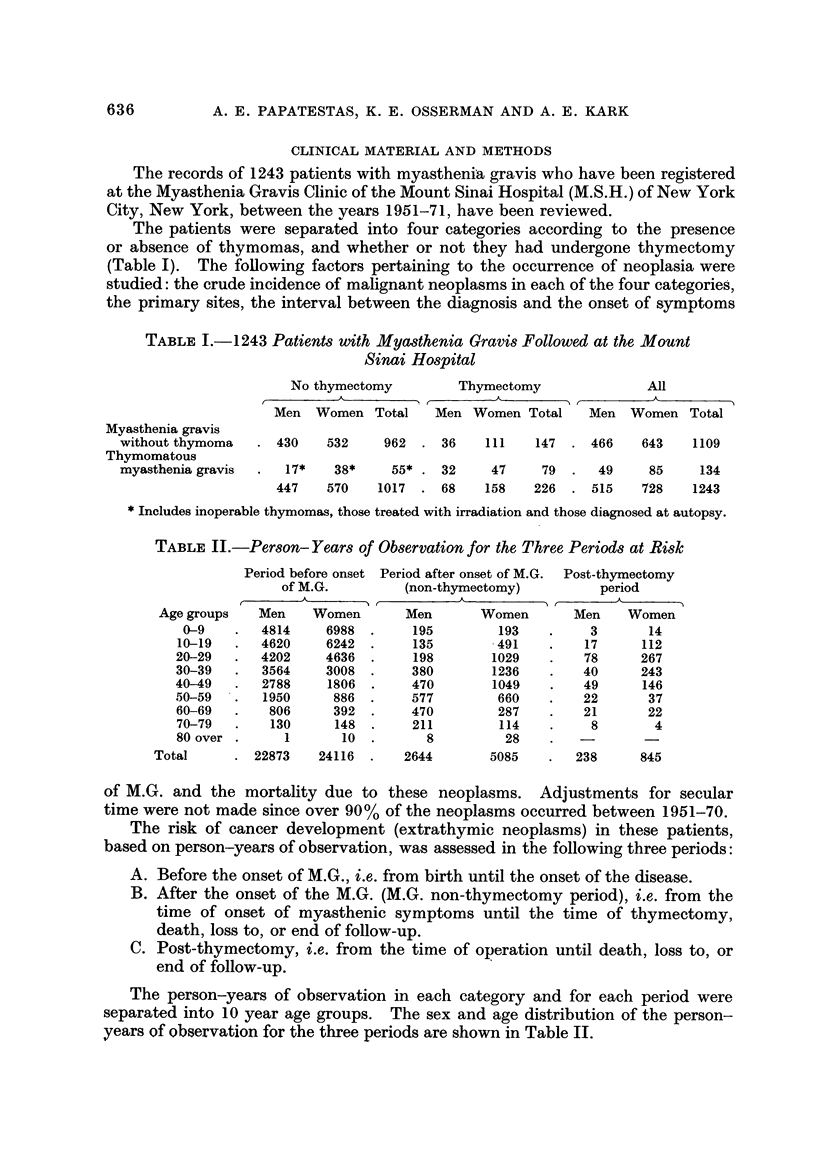

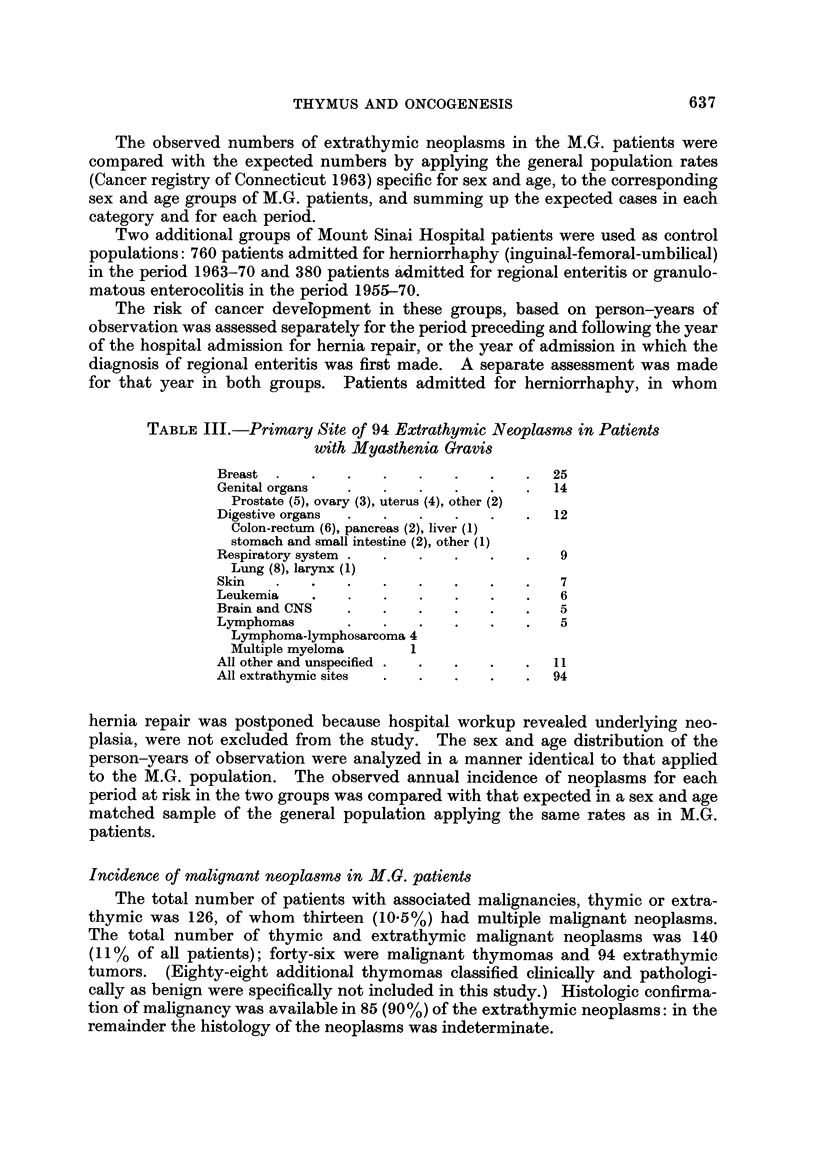

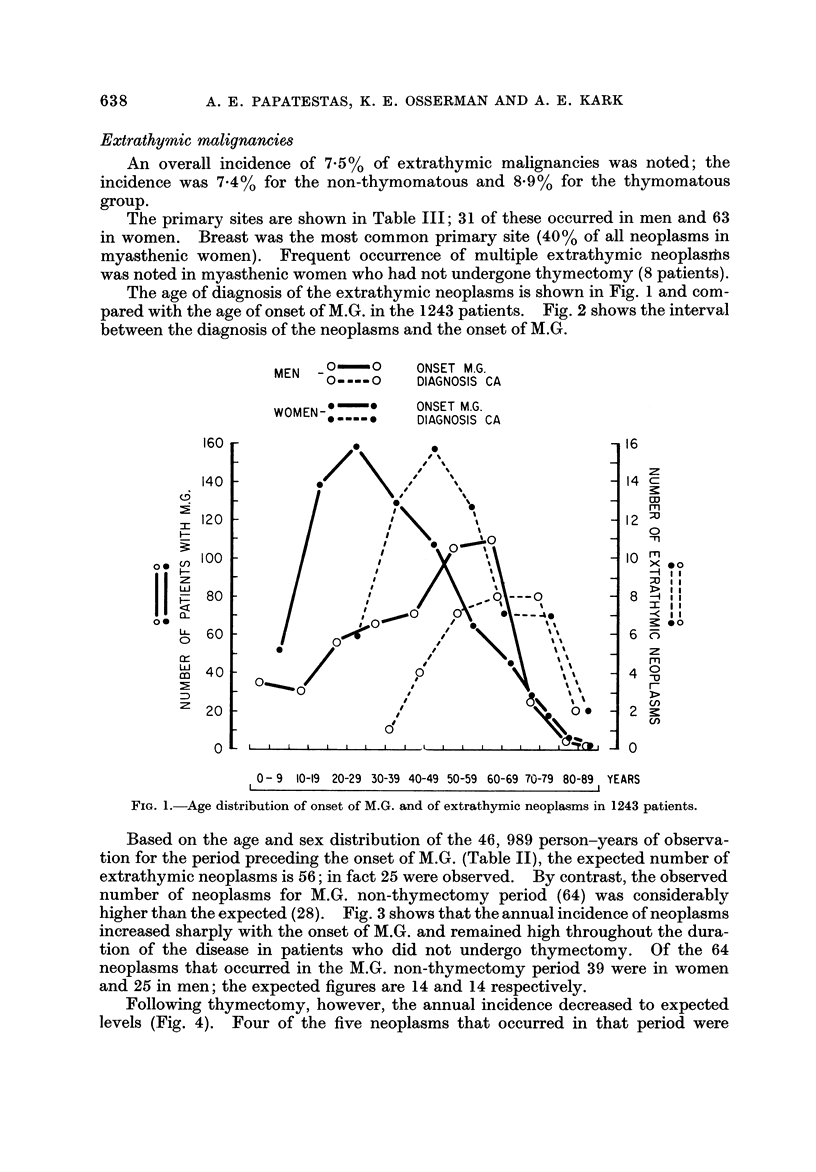

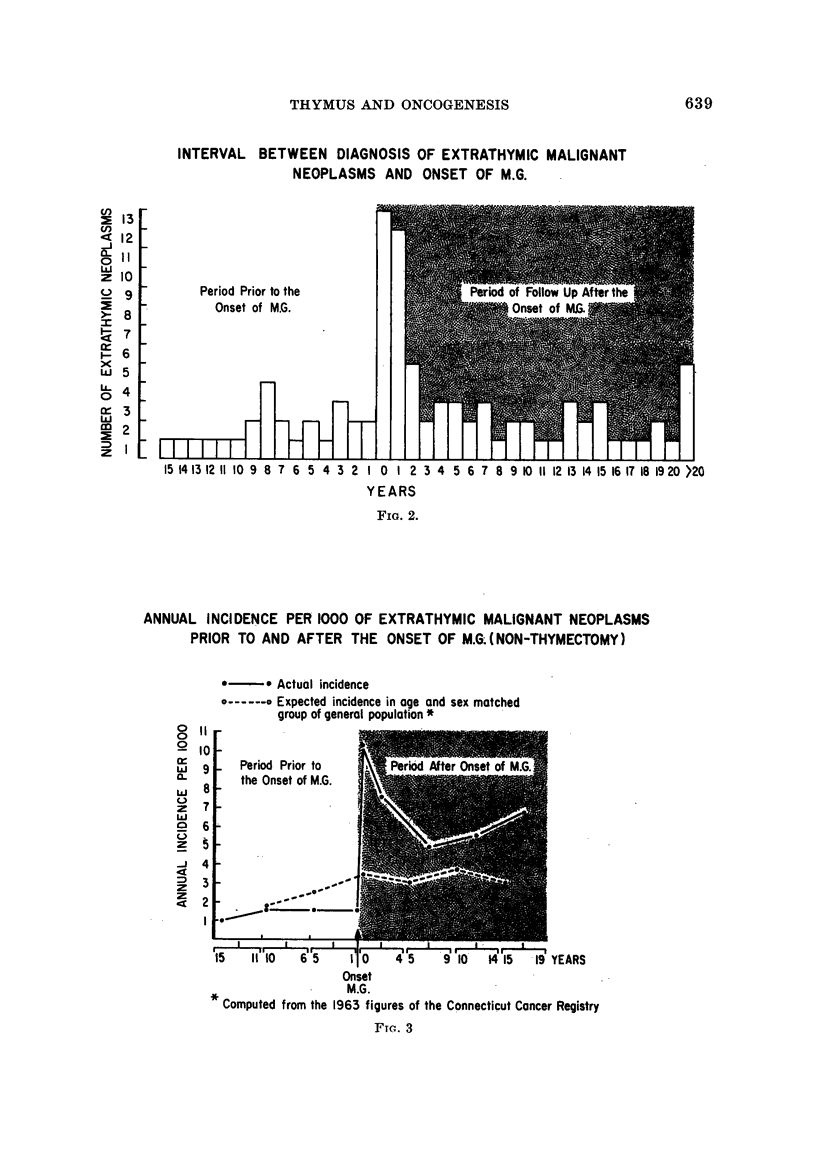

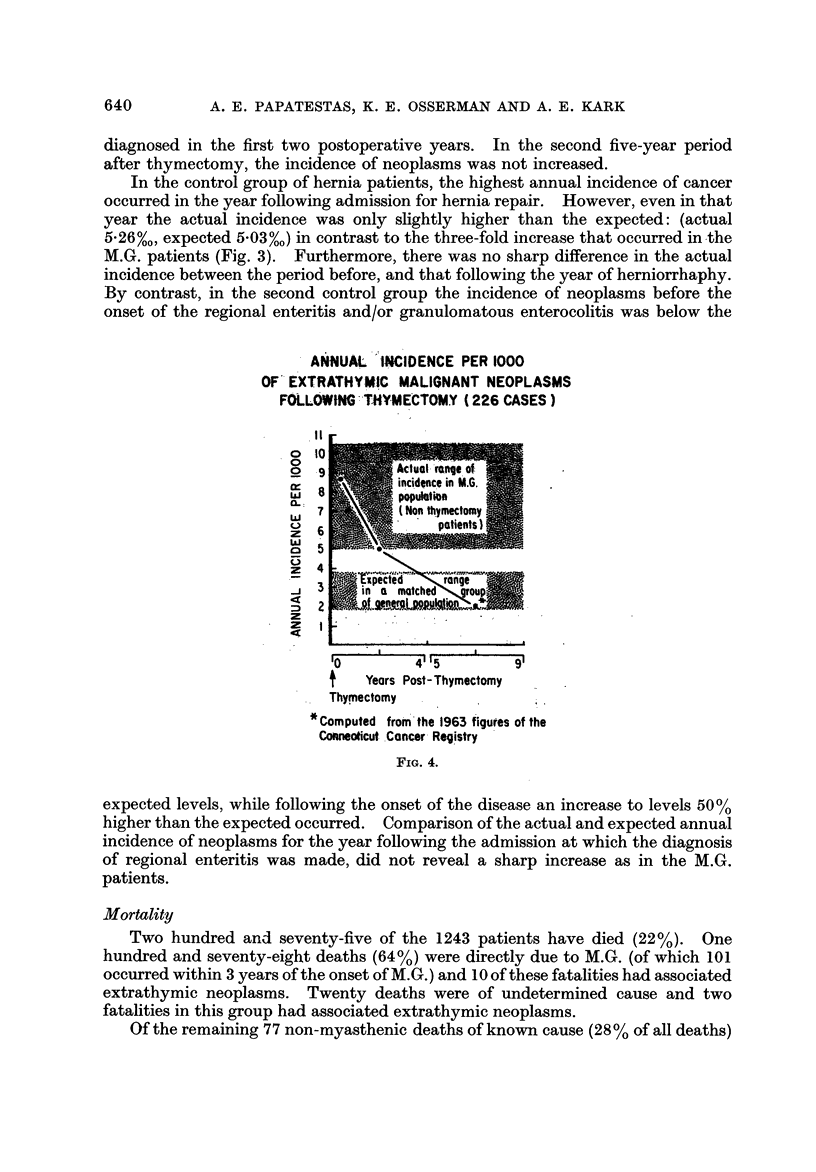

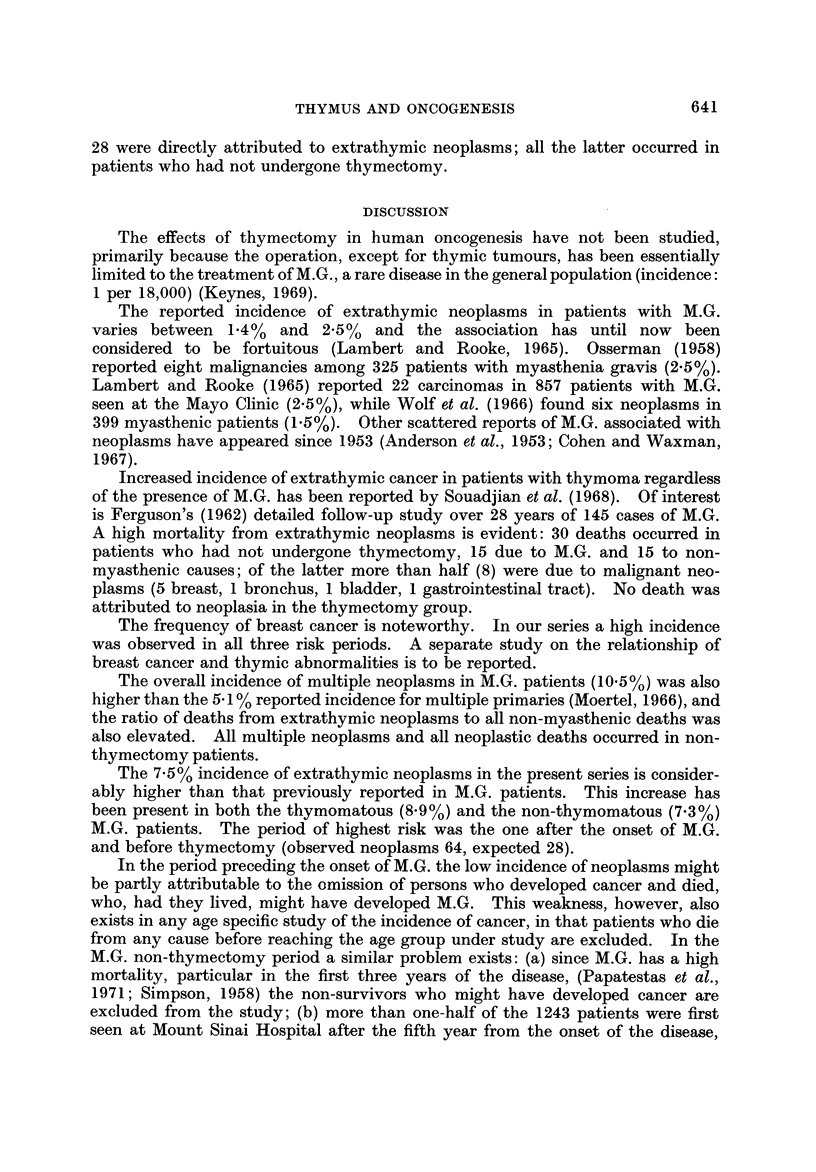

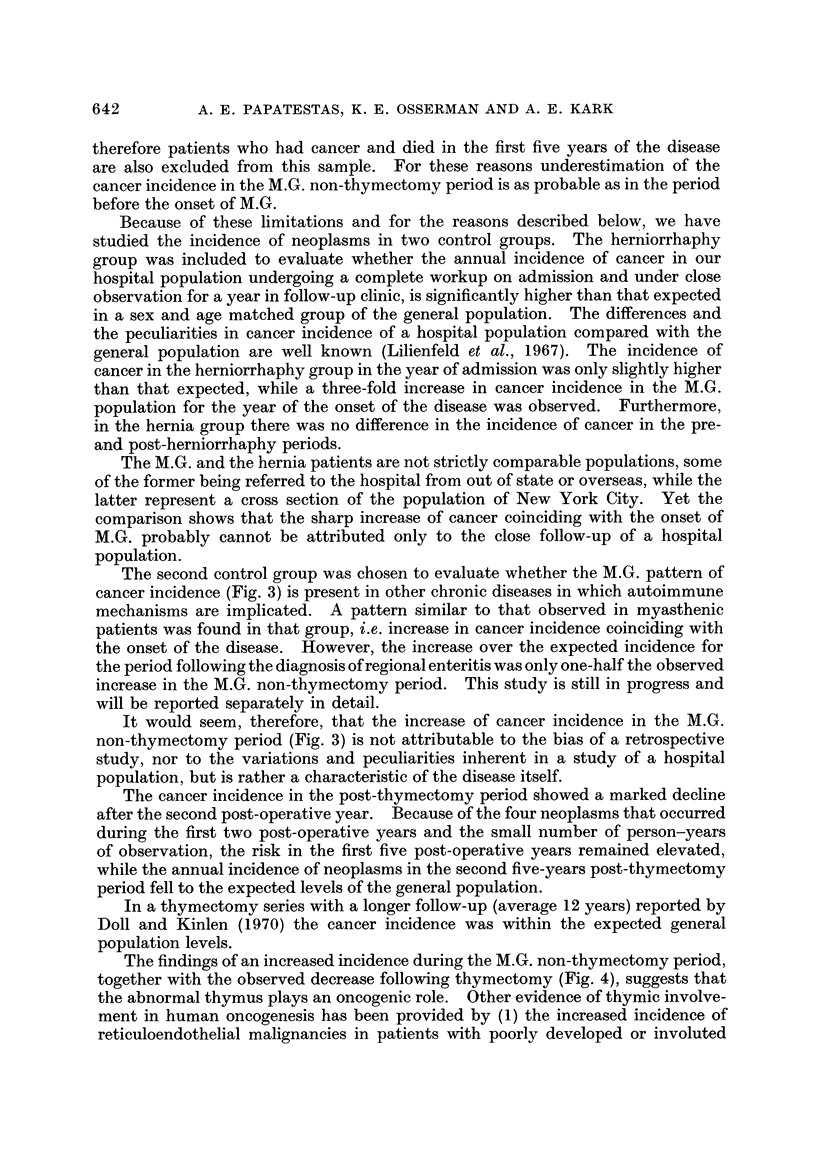

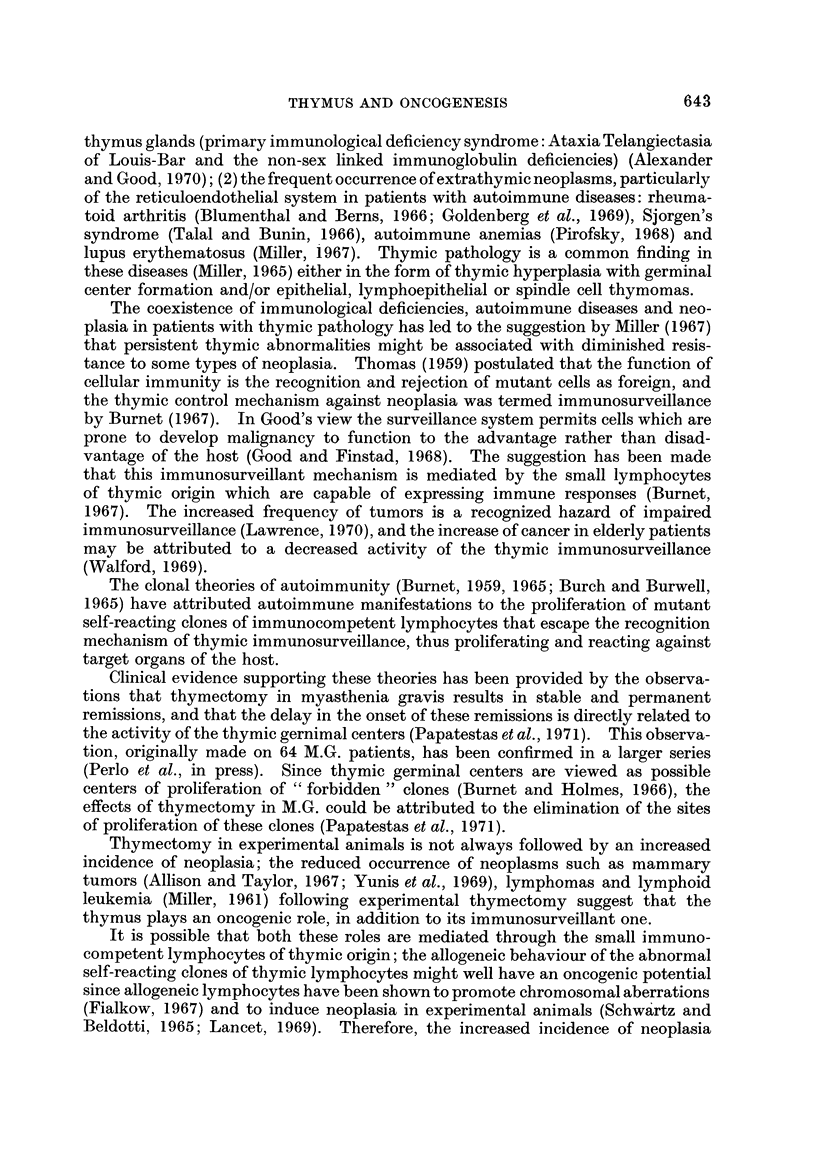

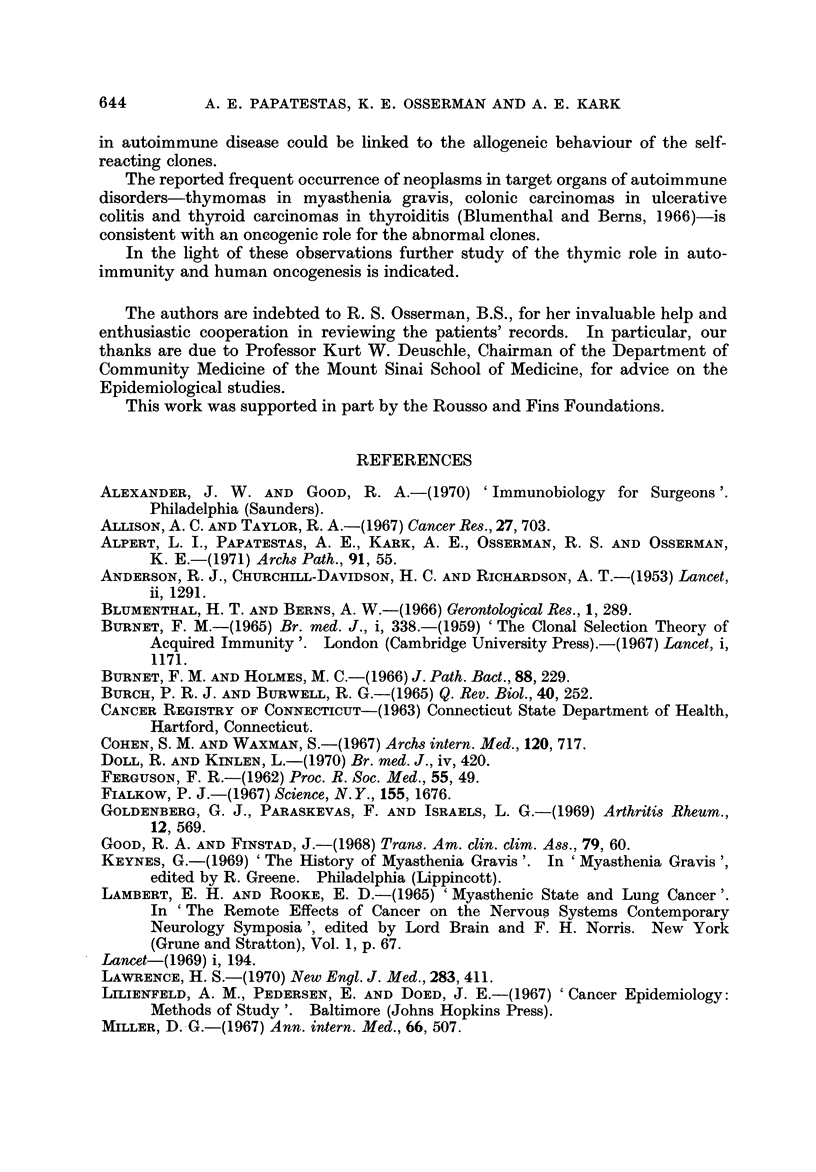

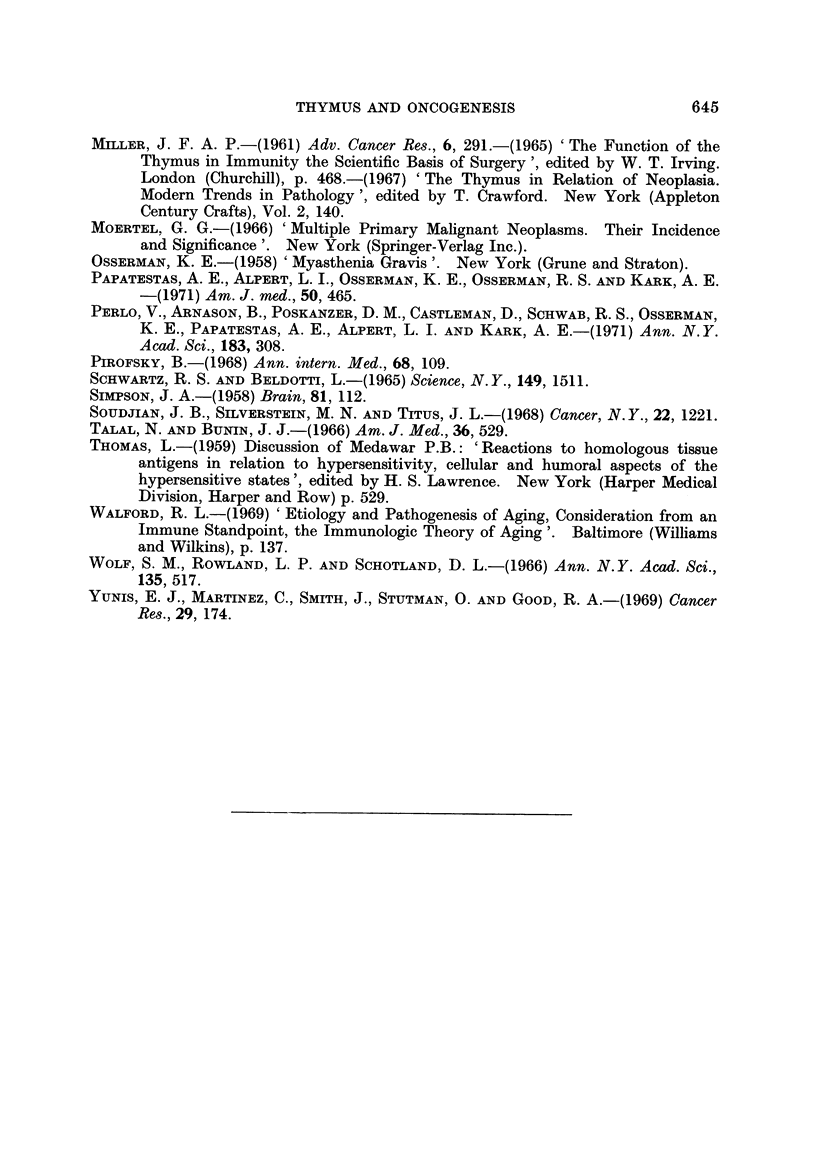


## References

[OCR_00765] Cohen S. M., Waxman S. (1967). Myasthenia gravis, chronic lymphocytic leukemia, and autoimmune hemolytic anemia. "A spectrum of thymic abnormalities?. Arch Intern Med.

[OCR_00771] Goldenberg G. J., Paraskevas F., Israels L. G. (1969). The association of rheumatoid arthritis with plasma cell and lymphocytic neoplasms.. Arthritis Rheum.

[OCR_00793] Miller D. G. (1967). The association of immune disease and malignant lymphoma.. Ann Intern Med.

[OCR_00806] Papatestas A. E., Alpert L. I., Osserman K. E., Osserman R. S., Kark A. E. (1971). Studies in myasthenia gravis: effects of thymectomy. Results on 185 patients with nonthymomatous and thymomatous myasthenia gravis, 1941-1969.. Am J Med.

[OCR_00815] Perlo V. P., Arnason B., Poskanzer D., Castleman B., Schwab R. S., Osserman K. E., Papatestis A., Alpert L., Kark A. (1971). The role of thymectomy in the treatment of myasthenia gravis.. Ann N Y Acad Sci.

[OCR_00820] SIMPSON J. A. (1958). An evaluation of thymectomy in myasthenia gravis.. Brain.

[OCR_00822] Souadjian J. V., Silverstein M. N., Titus J. L. (1968). Thymoma and cancer.. Cancer.

[OCR_00836] Wolf S. M., Rowland L. P., Schotland D. L., McKinney A. S., Hoefer P. F., Aranow H. (1966). Myasthenia as an autoimmune disease: clinical aspects.. Ann N Y Acad Sci.

[OCR_00840] Yunis E. J., Martinez C., Smith J., Stutman O., Good R. A. (1969). Spontaneous mammary adenocarcinoma in mice: influence of thymectomy and reconstitution with thymus grafts or spleen cells.. Cancer Res.

